# Examining the evidence for the health impact of combustion-free products: progress and prospects for tobacco harm reversal and reduction

**DOI:** 10.1007/s11739-021-02837-2

**Published:** 2021-09-15

**Authors:** Riccardo Polosa

**Affiliations:** 1 Institute of Internal Medicine, UOC Medicina Interna e Urgenza, AOU “Policlinico-V. Emanuele”, via S. Sofia, 78-Ed. 4, p. 2, stanza 78, 95100 Catania, Italy; 2 Department of Clinical and Experimental Medicine, University of Catania, Catania, Italy; 3 Center of Excellence for the Acceleration of Harm Reduction (CoEHAR), Università di Catania, Catania, Italy

**Keywords:** Heated tobacco products, E-cigarette, Smoking cessation, Switching trials, Tobacco harm reduction, Biomarkers

Chronic inhalational exposure to the mixture of more than 8000 harmful and potentially harmful chemical constituents that transfer into cigarette smoke during tobacco combustion can cause progressive functional alterations and structural damage in the human body, which result in numerous medical conditions affecting primarily the respiratory and cardiovascular systems [1, 2].

The key to reducing the negative health effects of smoking is to avoid chronic exposure to chemicals released during the tobacco combustion of conventional cigarettes. This can be achieved either by smoking cessation programs, which include prescription medications (varenicline, bupropion, nicotine replacement therapy) and counselling [3] or by cigarette substitution with combustion-free products, which include e-cigarettes and heated tobacco products (HTPs) [4].

Almost certainly smoking cessation programs and switching trials with non-combustible sources of nicotine would produce significant health improvements, thus confirming what might reasonably be predicted from what is already common knowledge on chemical composition of combustible cigarettes smoke and pathogenesis of smoking-related diseases. Switching from cigarettes to combustion-free products has been shown to improve respiratory outcomes in patients with COPD [5, 6] and to lower blood pressure in patients with hypertension [7]. Although these preliminary studies led to the expected results (i.e. that switching has similar clinical and functional benefits as quitting), their study design, sample size and reporting methods were not ideal.

To reassure health and regulatory authorities, larger prospective studies on the long-term health impact of combustion-free nicotine delivery products (C-F NDPs) are needed. It is essential to provide high quality clinical information on the harm reduction/reversal potential of CF-NDPs as a substitute for conventional cigarettes for long-term use; switching studies may form an integral part of regulatory submissions to the US Food and Drug Administration (FDA) when seeking authorization to market a novel product as a modified-risk tobacco product (MRTP) [8].

Gale et al. describe their approach to substantiating the potential for harm reduction/reversal of a newly marketed HTP in their carefully designed switching trial, published in *Internal and Emergency Medicine* [9].

The HTP device in their study (glo) electronically heats (instead of burning) small tobacco sticks at temperatures below 250 °C to generate nicotine-containing aerosols. Because these aerosols are produced at temperatures below combustion (which generally begins at temperature above 400 °C), they contain much less harmful and potentially harmful chemicals than tobacco smoke, and the overall level of chemical exposure in exclusive HTP users is considerably lower than in smokers [10]. Gale et al. executed the valuable scientific step of performing a reality check on the prediction that avoiding exposure to combustible cigarettes smoke by switching to much less harmful HTPs would almost certainly produce significant health improvements.

To verify this prediction, authors enrolled 506 participants in a 12-month randomised controlled study; smokers who did not intend to quit were randomised to either continue smoking cigarettes or switched to using the HTP device under investigation, while smokers who indicated they wanted to quit smoking were enrolled in a standard cessation program. In addition, a group of never smokers was also included as a reference group.

Subjects were tested monthly for “biomarkers of exposure” (to selected cigarette smoke toxicants) and “biomarkers of potential harm” (relative to oxidative stress, cardiovascular and respiratory disease, and cancer) including urine 11-dehydrothromboxane B2 (11-dTx B2), urine 8-epi-Prostaglandin F2α type III (8-Epi-PGF2α type III), white blood cell (WBC) count, plasma soluble intercellular adhesion molecule-1 (sICAM- 1), serum high-density lipoprotein (HDL), exhaled breath (FeNO), forced expiratory volume in 1 s (FEV_1_) and total 4‐(methylnitrosamino)‐1‐[3-pyridyl]‐1‐butanol (NNAL).

This study also included testing a biomarker called Acrylonitrile Haemoglobin Adduct N [2-cyanoethyl]valine (CEVal). CEVaL is an important compliance check, which identifies if participants in the switching and cessation arm of the study had recently smoked cigarettes [11]. Compliance to exclusive HTPs use and complete adherence to smoking cessation protocols is paramount in a study substantiating harm reduction when switching away from combustible cigarettes or when stopping smoking. Failure to completely replace or stop cigarettes would reduce or nullify the expected changes in biomarkers of exposure and potential harm. Specific CEVal threshold levels allowed authors to define the complete switchers and abstainers in which to assess biomarker changes.

Using data collected from 332 subjects between baseline and day 180, it was found that switching to HTPs not only reduced exposure to cigarette smoke toxicants, but also improved several BoPH when compared to continuing to smoke. In particular, the reported changes in 11-dTx B2, 8-Epi-PGF2α type III, WBC count, FeNO, and total NNAL (a potent pulmonary carcinogen in rodents) were directionally consistent with lessened health impact. In spite of the complete switch, the reported changes in BoPH, although statistically significant, were small, well within the variability of the tests, and as such of questionable clinical relevance. No significant changes were reported for sICAM- 1, HDL, and FEV1. The inclusion of a smoking cessation arm is critical to provide context to changes observed when participants switch from smoking to the HTPs use; similar small (and lack of) changes in BoPH were also observed in those undergoing smoking cessation over the same period of time. Moreover, another large switching trial of 984 smokers allocated to continue smoking cigarettes or to use an HTP device (IQOS) for 6 months showed modest (but yet clinically irrelevant) changes in HDL-C, WBC, FEV1, and total NNAL [12].

It is not surprising that there was no clinical impact. When quitting smoking, BoPH may improve, but the change may be gradual and may take years to become clinically relevant. Also, improvements in BoPH are most likely to occur in smokers who have already been affected by pathophysiological changes and structural damage. For example, no significant changes in pulmonary function tests have been ever reported in smokers with normal spirometry at baseline who quit after switching to e-cigarettes, compared to those continuing to smoke [13, 14]. Not to forget, the relatively short 6-month follow up of the study by Gale et al. is insufficient to show meaningful smoking cessation responsive BoPH improvements and a much longer period of observation is necessary. Final results will not be available until the end of 2021, when the 12-month results will be released. Once we can determine whether the reduction in BoPH is maintained throughout the study, we will also be able to fully appreciate the magnitude of harm reduction potential that may be achievable by switching exclusively to HTPs.

Lack of sensitive and clinically significant endpoints for efficient substantiation of smoking cessation and tobacco harm reduction/reversal has been long acknowledged [15]. Reframing health effect indicators that are specifically relevant to smoking cessation and switching trials is the logical next step; several promising candidate outcome measures should be considered. For the FDA, it is useful to explore the health effects of novel tobacco and nicotine products to better understand their impact, in addition to examining additional study endpoints for the evaluation of the short- and long-term effects in clinical studies [16].

At the University of Catania’s Center of Excellence for the Acceleration of Harm Reduction (CoEHAR), researchers are carefully examining a number of relatively unexplored indicators of health effects for the best possible detection of biological/physiological changes associated with stopping smoking and for use in regulatory science. These included imaging studies (e.g. high-resolution computed tomography, hyperpolarized magnetic resonance imaging, optical coherence tomography angiography, quantitative light-induced fluorescence scanning), digitized patient-reported outcomes (e.g. App-based questionnaires) as well as functional tests (e.g. impulse oscillometry, forced expiratory flow from 25 to 75% of vital capacity, maximal oxygen consumption, augmentation index, pulse wave velocity, mucociliary clearance transit time).

Results so far are promising and generally supportive of their value. For example, by measurement of mucociliary clearance transit time (MCCTT) researchers can detect early respiratory health changes in smoking cessation studies and switching trials. Chronic exposure to cigarette smoke is known to cause progressive structural damage and functional alterations of the lungs, with loss of cilia, reduced ciliary beating, and airway epithelial mucus cell hyperplasia with mucus hypersecretion [17, 18]. Functional impairment and structural damage of the respiratory tract contribute to prolonged mucociliary clearance transit time (MCCTT) in smokers [19, 20] and stopping smoking may quickly reduce structural damage and restore cilia-mucus interaction [21, 22]. In a recent study we have shown that smokers who stopped smoking by switching to exclusive use of combustion-free nicotine delivery systems (such as ECs and HTPs) exhibited similar MCCTT as never and former smokers (23). It appears that MCCTT restoration occurs relatively soon after quitting. As well as being relevant to the investigation of possible health issues, the study’s conclusions could be very valuable in assisting with the substantiation of harm reduction and reversal of adverse effects of combustion-free nicotine/tobacco products.

Considering that many smokers continue to smoke despite the health risks, combustion-free nicotine delivery technology has enormous potential to help reduce smoking rates. Researchers should keep committing to high quality research with the goal of accurately delineating the harm reduction potential of these new technologies and advancing the current understanding of their impact on human health.
